# Factors affecting the growth of infants diagnosed with cystic fibrosis by newborn screening

**DOI:** 10.1186/s12887-019-1727-9

**Published:** 2019-10-15

**Authors:** K. D. Patterson, T. Kyriacou, M. Desai, W. D. Carroll, F. J. Gilchrist

**Affiliations:** 1 Institute of Science and Technology in Medicine, Keele University, Stoke-on-Trent, ST4 7QB UK; 2 School of Computing and Mathematics, Keele University, Keele, ST5 5BG UK; 3 Department of Paediatric Respiratory Medicine, Birmingham Children’s Hospital, Steelhouse Lane, Birmingham, B4 6NH UK; 4 Paediatric Respiratory Services, Royal Stoke University Hospital, University Hospitals of North Midlands NHS Trust, Stoke on Trent, ST4 6QG UK; 5 Institute of Applied Clinical Science, Keele University, Stoke-on-Trent, ST4 7QB UK

## Abstract

**Background:**

Newborn screening (NBS) for cystic fibrosis (CF) improves nutritional outcomes. Despite early dietetic intervention some children fail to grow optimally. We report growth from birth to 2 years in a cohort of children diagnosed with CF by NBS and identify the variables that influence future growth.

**Methods:**

One hundred forty-four children were diagnosed with CF by the West Midlands Regional NBS laboratory between November 2007 and October 2014. All anthropometric measurements and microbiology results from the first 2 years were collated as was demographic and CF screening data. Classification modelling was used to identify the key variables in determining future growth.

**Results:**

Complete data were available on 129 children. 113 (88%) were pancreatic insufficient (PI) and 16 (12%) pancreatic sufficient (PS). Mean birth weight (z score) was 3.17 kg (− 0.32). There was no significant difference in birth weight (z score) between PI and PS babies: 3.15 kg (− 0.36) vs 3.28 kg (− 0.05); *p* = 0.33. By the first clinic visit the difference was significant: 3.42 kg (− 1.39) vs 4.60 kg (− 0.48); *p* < 0.0001. Weight and height remained lower in PI infants in the first year of life. In the first 2 years of life, 18 (14%) infants failed to regain their birth weight z score. The median time to achieve a weight z score of − 2, − 1 and 0 was 18, 33 and 65 weeks respectively. The median times to reach the same z scores for height were 30, 51 and 90 weeks. Birth weight z score, change in weight z score from birth to first clinic, faecal elastase, isolation of *Pseudomonas aeruginosa*, isolation of *Staphylococcus aureus* and sweat chloride were the variables identified by the classification models to predict weight and height in the first and second year of life.

**Conclusions:**

Babies with CF have a lower birth weight than the healthy population. For those diagnosed with CF by NBS, the weight difference between PI and PS babies was not significantly different at birth but became so by the first clinic visit. The presence of certain factors, most already identifiable at the first clinic visit can be used to identify infant at increased risk of poor growth.

## Background

The implementation of newborn screening (NBS) has fundamentally changed the care of children with cystic fibrosis (CF) [1]. They are now identified in the first few weeks of life and no longer have to wait for the presenting symptoms to become severe enough to warrant investigation [2]. Historically, this wait was often associated with failure to thrive and irreversible lung damage [3]. CF-NBS improves outcomes, particularly nutritional ones [4, 5]. This is important for children with CF as nutritional status is closely related to lung function, quality of life and ultimately survival [6]. The nutritional benefits of CF-NBS are maintained into adulthood [7].

Despite early diagnosis and the initiation of appropriate treatment, some infants diagnosed with CF by NBS do not achieve optimal growth [8]. Antenatal factors such as maternal smoking or poor nutrition can contribute to this, as can postnatal factors such as acute or chronic respiratory infections, adherence with therapy, nutritional intake, dose of pancreatic enzyme replacement therapy and other environmental exposures [9, 10]. It would be useful for clinicians to know which variables have the most significant impact on infant growth. This would mean children at higher risk of growth failure could be identified and considered for more careful monitoring of growth and/or earlier nutritional support. We collated NBS, demographic, microbiology and growth data on a cohort of children diagnosed with CF by NBS. We then used classification modelling to establish the factors / variables useful in predicting infant growth.

## Method

### Study population

A cohort of 144 children diagnosed with CF by NBS was identified. This included all babies with a positive CF NBS from the West Midlands Regional Screening Laboratory between 1st November 2007 and 31st October 2014 who had a confirmed diagnosis of CF (identification of two CF-causing mutations and/or a sweat chloride ≥60 mmol/L). Five children categorized as Cystic Fibrosis Screen Positive Inconclusive Diagnosis (CF SPID) [11] were excluded. Two children had died and two moved out of area. A further six patients were excluded due to incomplete datasets. The remaining 129 children attended one of the two tertiary CF centres in the region: Birmingham Children’s Hospital and Royal Stoke University Hospital. Although these two centres work independently, both follow the UK CF Trust and European CF Society Standards of care resulting in similar clinical practices [12, 13].

### Data collection

Demographic and newborn screening data were collected. These included date of diagnosis (taken as date of first clinic visit or date of diagnosis of meconium ileus), gender, ethnicity, birth weight, the presence of meconium ileus, mean immunoreactive trypsinogen, CFTR mutations, sweat chloride, faecal elastase (measured at first clinic appointment), weight at first clinic visit and mode of feeding (exclusive breast / exclusive formula / mixed). All weight and length measurements recorded in the first 2 years of life were then collated. Insufficient data points were available for head circumference to allow meaningful analysis. We also recorded age at first isolation of *Pseudomonas aeruginosa* and *Staphylococcus aureus.* These data were obtained from the West Midlands NBS Laboratory database, patient’s clinical notes and the electronic results systems at the two CF Centres. All height and weight measurements were converted into z scores using the WHO-UK anthropometric calculator. This corrects for sex and gestational age. To summarise growth, the following four outcomes were calculated for each child: mean weight z score in first year of life, mean weight z score in second year of life, mean height z score in the first year of life and mean height z score in second year of life. The median time for children to achieve height and weight z scores of − 2, − 1 and 0 was calculated and children who did not regain their birth weight z score in the first 2 years of life were identified.

### Classification modelling to identifying variables affecting growth from 0 to 2 years

Before classification modelling could be undertaken, the cohort had to be validated using a clustering algorithm (k-means) [14]. This ensured it was representative of the UK CF population. The anthropometric measurements as well as the demographic, newborn screening and microbiology data were included as inputs and the mathematically generated clusters were scrutinised by the CF clinicians (MD, WC and FG) to ensure they corresponded to clinical phenotypes. Decision-tree classification models were then generated to determine the variables that predicted future growth. Inputs were the demographic, newborn screening and infection variables. The model analyses all the inputs but only uses those with predictive effect in the decision trees. The four outcomes were the mean z scores for height and weight in the first and second year of life. These outputs were chosen as the z scores frequently varied significantly over time meaning a z score from a single time point would not have been representative. Each classification model was validated using a stratified five-fold cross-validation method. This approach is used when the sample size does not allow for a representative split (such as 70% / 30%) for model training and validation sets. The data is split into five sets ensuring each set has proportional representation of all classes in each set. Modelling is repeated five times each using a different fifth of the data for validation. The final model reported is the one obtained using all the data, however, the error reported is the mean error out of the five repeats.

## Results

### Patient demographics

The sample included 63 (49%) girls and 66 (51%) boys. Mean (SD) gestation age was 38.9 (1.8) weeks with 8 (6%) born < 37 weeks gestation. Twenty one (16%) children had meconium ileus. Median (IQR) age at diagnosis was 22 (17–25) days. This was later in pancreatic sufficient (PS) compared to pancreatic insufficient (PI) babies: 25.5 (21.5–32) vs 20 (16–25), *p* = 0.004. 121/129 (94%) children were Caucasian and 8/129 (6%) were Asian, Afro-Caribbean or other white. 68/129 (53%) were homozygous and 122/129 (95%) heterozygous for the Phe508del variant.

### Nutrition and effect of pancreatic function

The mean (SD) birth weight of the entire cohort was 3.17 (0.51) kg giving a mean (SD) z score of − 0.32 (1.13). The mean (SD) birth weight was 3.21 (0.50) kg for boys and 3.12 (0.53) kg for girls. The mean (SD) birth weight z scores were − 0.35 (1.07) for boys and − 0.30 (1.20) for girls. Using a faecal elastase cut-off of 200 μg/g, 113 (88%) children were PI and 16 (12%) PS. When reviewed at their first clinic visit, 29 (22%) infants were exclusively breast-fed, 60 (47%) were exclusively formula milk and 40 (31%) were mixed feeding. There was no difference in mode of feeding between PI and PS. All babies were started on standard treatment on the day of diagnosis. The difference in weight between PI and PS infants was not significant at birth but became so by the time of the first clinic visit. Height and weight was significantly lower in PI children in the first year of life. The difference was less in the second year and no longer significant. See Table 1. In the first 2 years of life, 18 (14%) of infants failed to regain their BW z score, all of these were pancreatic insufficient. The median time for children to achieve a weight z score of − 2, − 1 and 0 was 18, 33 and 65 weeks respectively. The median times to reach the same z scores for height were 30, 51 and 90 weeks.

**Table 1:** Weight at birth and first clinic visit for pancreatic sufficient and insufficient infants

	Pancreatic insufficient (*n* = 113)	Pancreatic sufficient (*n* = 16)	*p* value
Birth weight			
kg	3.15 (0.51)	3.28 (0.53)	0.33
z score	−0.36 (1.11)	−0.05 (1.26)	0.29
First clinic weight			
kg	3.42 (0.80)	4.60 (1.49)	< 0.0001
z score	−1.39 (1.27)	−0.48 (1.62)	0.005
Change in weight z score from birth to first clinic			
Absolute	−1.03 (0.81)	−0.43 (1.06)	0.023
Rate	−0.35 (0.28)	−0.10 (0.35)	0.0007
Weight in year 1			
z score	−0.73 (1.27)	− 0.25 (1.16)	< 0.0001
Weight in year 2			
z score	−0.16 (0.93)	0.37 (1.02)	0.191
Height in year 1			
z score	−0.67 (1.29)	−0.12 (1.18)	0.001
Height in year 2			
z score	−0.37 (1.03)	−0.19 (1.00)	0.255

All data is presented as mean (SD). Rate: change per week

*kg* kilograms

### Microbiology data

All the children were on oral *Staphylococcus aureus* prophylaxis. *Staphylococcus aureus* was isolated in 16 of the 129 children in the first 2 years of life. The median (IQR) age of the first isolate was 0.22 (0.08–0.63) years. *Pseudomonas aeruginosa* was isolated in 45 children in the first 2 years of life. The median (IQR) age of the first isolate was 1.18 (0.82–1.49) years. All these isolates were non-mucoid. No child met the Leeds Criteria for chronic *Pseudomonas aeruginosa* infection in the first 2 years of life [15].

### Variables affecting future growth

**Fig. 1:**
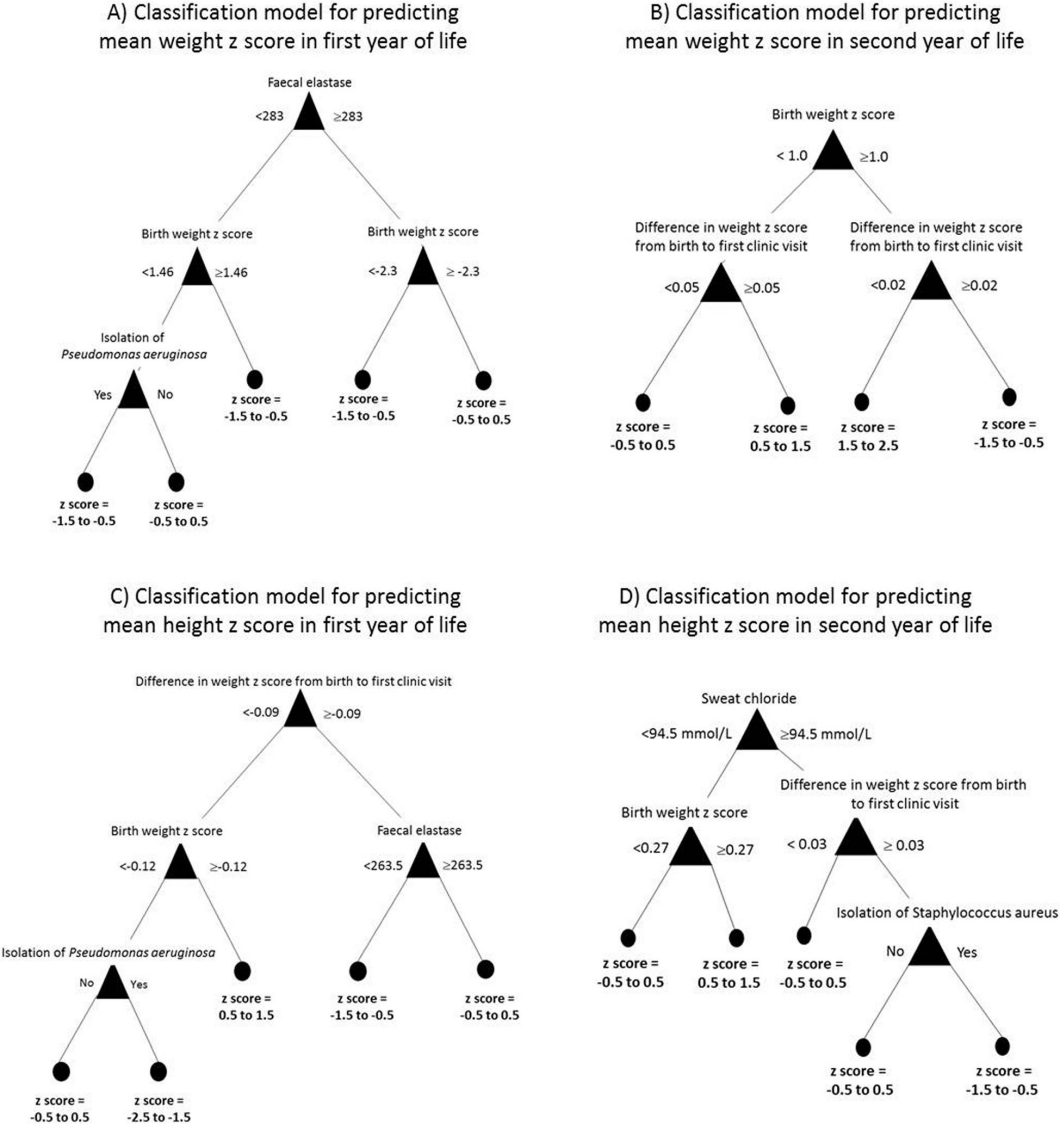
Decision tree models generated by classification modelling showing the factors predictive of mean weight and height z scores in the first and second year of life

The unsupervised cluster analysis identified two distinct groups. See Table 2 in Appendix. The CF clinicians agreed the clusters were clinically relevant as they represented PI and PS phenotypes. This validated the cohort. Decision tree classification modelling was therefore undertaken to identify the variables that could be used to predict the growth outcomes listed above. See Fig. 1. Across the four models, birth weight z score, change in weight z score from birth to first clinic, faecal elastase, isolation of *Pseudomonas aeruginosa*, isolation of *Staphylococcus aureus* and sweat chloride were the variables of use in predicting future growth. Importantly, the mode of feeding was not identified as a predictor of infant growth by any of the classification models.

## Discussion

This study reports growth from birth to 2 years in a cohort of children diagnosed with CF by NBS. Classification modelling is also used to identify the variables affecting infant growth, to our knowledge this it is the first time this methodology has been used in this group.

Previous cohort studies show CF babies have a lower birth weight than healthy children although it is usually still within the normal range [16, 17]. Our cohort follows this pattern with a mean birth weight z scores of − 0.32. The lower birth weight may relate to gestational age as this was on average 38.9 weeks. Although the WHO-UK anthropometric calculator corrects the birth weight z scores for gestational age, it only does this for those < 37 weeks. CFTR may have a role in prenatal growth. As children with PS CF have higher residual CFTR function than PI CF infants, this would be consistent with the observation of lower birth weights in PI infants. This hypothesis requires replication in a larger cohort as the difference in birth weight between PI and PS infants was not statistically significant. Interestingly the gestation age was very similar for PI and PS infants (38.5 versus 38.8 weeks). Infants with CF have been shown to have reduced levels of insulin like growth factor [18]. This may explain the difference in birth weight between CF and non-CF babies and why some children with well managed CF still fail to reach their growth potential.

Postnatally, pancreatic function has a clear effect with the weight difference between the PS and PI infants becoming statistically significant at the first clinic visit when the child had ‘untreated CF’ for an average of 22 days. The degree of faltering growth observed in this short period was one of the most important variables identified by the classification model in predicting infant growth. Therefore, infants who experience the most severe nutritional consequences before treatment, continue to do so after this is started. These infants are likely to be those with mutations associated with the most severe loss of CFTR function. Despite treatment with pancreatic enzyme replacement therapy, PI infants had a significantly lower height and weight in the first year of life than PS infants. This difference reduced in the second year showing catch-up growth can be achieved with prolonged treatment pancreatic enzyme replacement therapy and appropriate nutritional support.

Infants with CF who are PI are more likely to have lower weight and height z scores than those who are PS [16]. It is therefore unsurprising that faecal elastase is one of the variables used by the classification models to predict future growth. PI infants usually have at least one class I, II or III mutation which are associated with higher sweat chloride which explains its inclusion in some of the models. Chronic infection with PA has an adverse effect on nutritional parameters in children with CF [17]. In our study, a single isolation of PA was linked with reduced infant weight and height. It is possible this was a direct effect of the PA infection or more likely that children who isolate PA have more significant lung disease which affects growth. The effects of acute and chronic SA infection on the growth of children in CF is not well known but early infection, especially occurring as a co-infection with other organisms, may be associated with severe lung disease which would affect growth [19, 20].

The importance of birth weight in predicting future growth is well established in the healthy children. The GECKO Drenthe Birth Cohort included 2447 healthy infants and identified birth weight and mode of feeding as the most important factors at predicting growth in the first 6 months of life [18]. The classification modelling in our study did not identify mode of feeding as a factor predictive of future growth although the sample size may have been too small to identify this. Early nutritional status is a key predictor of future health in children with CF [20]. It is therefore unsurprising that CF children with low birth weight have increased pulmonary disease in later childhood [17]. Increasing the birth weight of children with CF would therefore have long term health benefits. This could only be achieved through general public health measures targeting all pregnant mothers as it is unusual for the diagnosis of CF to be made prenatally.

Our study has several limitations. As we did not have a matched control group of non-CF infants from the same area we relied on the WHO-UK anthropometric calculator to generate z scores but accept this may miss local variations. There are potentially a huge number of variables that could affect infant growth. Due to the retrospective nature of this study we were limited as to which factors we could collect data on. Relevant variables which we were unable to collect data on included: parental height and weight, maternal smoking status, maternal diet during pregnancy, pulmonary exacerbations, treatment regimen, nutritional support, adherence with treatment and socioeconomic status. All of these may affect infant growth [9]. We used mean z scores over the first and second years of life for height and weight as our outcome for the statistical modelling. We accept this approach lacks granularity but the wide variation seen in the individual measurements it gave us the most useful summary outcome. We considered using alternative growth indices calculated from the weight and height measurements. However, the WHO does not recommend using BMI for age in children under 2 years and as percentage weight-for-height is not independent of the raw data it would have made the modelling results difficult to interpret.

## Conclusion

This cohort study confirms children with CF have a lower birth weight than non-CF babies. The effect of pancreatic insufficiency on weight was not significant at birth but became so by the first clinic visit and remained so for the first year of life. A number of CF babies did not regain their birth weight z score by 2 years of age. Identification of predictors of poor infant growth such as low birth weight and poor weight gain from birth to first clinic should enable focused interventions by clinicians and dieticians to optimise nutritional outcomes. The possible effect of CFTR on antenatal growth and the role of other factors such as adherence to treatment and socioeconomic status on infant growth warrants further investigation.

## Data Availability

The datasets used during the current study are available from the corresponding author on reasonable request.

## Appendix

**Table 2:** Centroid values from the unsupervised cluster analysis used to validate the dataset

Variable	Cluster 1 (members: 113)	Cluster 2 (members: 16)
Birth weight z score	−0.42	−0.72
Change in weight z score from birth to first clinic visit	0.00	0.01
Faecal Elastase	15	466
Sweat chloride	99	46.5
Immunoreactive trypsinogen	172	85.5
CFTR Mutation 1	Phe508del	Phe508del
CFTR Mutation 2	Phe508del	Arg117His
Gestation	39	39.5
Mean length z score in year 1	−0.68	−0.14
Mean length z score in year 2	−0.24	− 0.12
Mean weight z score in year 1	−0.67	− 0.04
Mean weight z score in year 2	0.18	0.19

All data presented as mean apart from genotype which indicates the most prevalent mutation

## Abbreviations

CF SPID: Cystic fibrosis screen positive inconclusive diagnosis
CF: Cystic fibrosis
CFTR: Cystic fibrosis transmembrane conductance regulator
IQR: Interquartile range
NBS: Newborn screening
PI: Pancreatic insufficient
PS: Pancreatic sufficient
SD: Standard deviation
